# Mammal and tree diversity accumulate different types of soil organic matter in the northern Amazon

**DOI:** 10.1016/j.isci.2023.106088

**Published:** 2023-01-30

**Authors:** María Losada, Antonio M. Martínez Cortizas, Kirsten M. Silvius, Sara Varela, Ted K. Raab, Jose M.V. Fragoso, Mar Sobral

**Affiliations:** 1 CRETUS - EcoPast (GI-1553), Departmento de Edafoloxía e Química Agrícola, Facultade de Bioloxía, Universidade de Santiago de Compostela, 15782 Santiago de Compostela, Spain; 2 Bolin Centre for Climate Research, Stockholm University, 106 91 Stockholm, Sweden; 3 Department of Forest Resources and Environmental Conservation, Virginia Tech University, Blacksburg, VA 24061, USA; 4 MAPAS Lab, Departamento de Ecoloxía e Bioloxía Animal, Universidade de Vigo, 36310 Vigo, Spain; 5 Carnegie Institution for Science, Deparment of Global Ecology, Stanford, CA 94305, USA; 6 Departamento de Zoologia, Universidade de Brasılia, Brasılia, DF 70910-900, Brazil; 7 Institute of Biodiversity Science and Sustainability, California Academy of Sciences, San Francisco, CA 94118, USA

**Keywords:** Environmental science, Ecology, Biological sciences

## Abstract

Diversity of plants and animals influence soil carbon through their contributions to soil organic matter (SOM). However, we do not know whether mammal and tree communities affect SOM composition in the same manner. This question is relevant because not all forms of carbon are equally resistant to mineralization by microbes and thus, relevant to carbon storage. We analyzed the elemental and molecular composition of 401 soil samples, with relation to the species richness of 83 mammal and tree communities at a landscape scale across 4.8 million hectares in the northern Amazon. We found opposite effects of mammal and tree richness over SOM composition. Mammal diversity is related to SOM rich in nitrogen, sulfur and iron whereas tree diversity is related to SOM rich in aliphatic and carbonyl compounds. These results help us to better understand the role of biodiversity in the carbon cycle and its implications for climate change mitigation.

## Highlights

• Soil organic matter (SOM) varied in carbonyl and aliphatic groups versus N, S, Fe content
• Tree diversity relates to the accumulation of aliphatic and carbonyl SOM
• Mammal diversity contributes to the accumulation of N-, S-, Fe-rich SOM
• Mammals and trees’ effect on SOM composition is complementary, thus not redundant

## Graphical abstract

**Figure:**
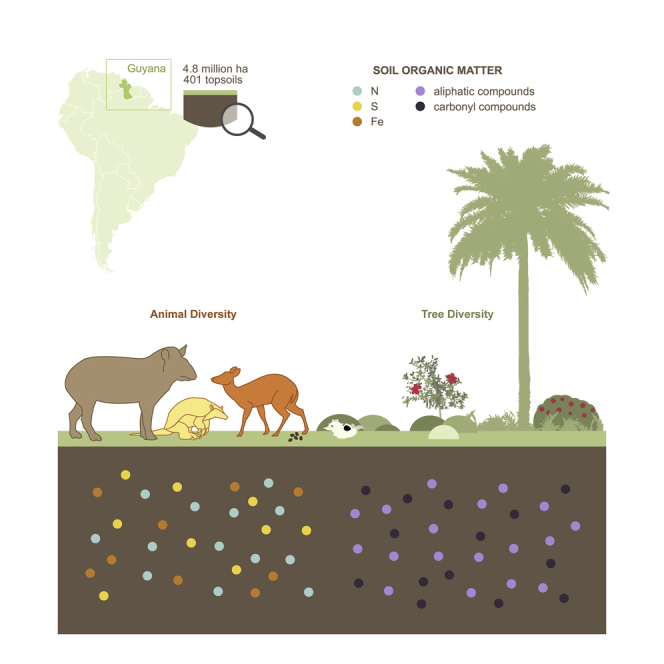

## Introduction

Living organisms—plants, animals, and microbes—shape soil structure, alter nutrient transfer, and regulate decomposition and mineralization of soil organic matter (SOM).^1^^,^^2^^,^^3^^,^^4^ SOM is composed of molecules based on carbon. Plants are well documented to contribute to SOM, and thus soil carbon.^5^^,^^6^^,^^7^ More recently, it has been reported that mammals also affect carbon concentration in soils by generating organic remains through trophic interactions with plants.^8^

Mammals and other animals influence soil composition in many ways: First, they increase SOM through their inputs of fecal matter but also through carcasses and excretory compounds.^9^ Second, animal remains dramatically change the diversity^10^ and activity of the soil microbial community.^11^ Third, animals transport and homogenize plant- and animal-derived materials,^12^ but they can also increase heterogeneity and quantity of nutrient inputs to soil.^13^^,^^14^^,^^15^ Last, animals modify the structure and species composition of tree communities,^16^^,^^17^^,^^18^ which in turn can alter inputs to the SOM and soil carbon accumulation.

Soil organic carbon (SOC) accumulation depends on the origin (animal, plant, or microbial), composition and molecular diversity of organic inputs in soil.^19^ Not all carbon forms are equally resistant to mineralization by microbes and thus, relevant to carbon storage.^20^^,^^21^^,^^22^ Thus, whether and to what extent animal diversity, interacting with plant diversity, actually affects SOC, will depend on the composition of SOM accumulated through animal activities.^23^ Labile carbon forms such as polysacharides and proteins are rapidly and easily mineralized through microbial respiration into CO_2_,^24^ whereas recalcitrant carbon molecules such as lignin, O-alkyl and aliphatic compounds, are slowly and less efficiently degraded by microbes, accumulating in soil as stable carbon forms.^25^^,^^26^ Plant-derived organic compounds are primarily polysaccharides, i.e., labile carbon-rich SOM, and lignin, i.e., recalcitrant carbon-rich SOM.^27^ In contrast, animal feces and urine provide nitrogen inputs to soil^14^^,^^28^ and their carcasses are a varying source of amino acids and amonium.^29^^,^^30^^,^^31^ For example, keratins are sulfur-rich fibrous proteins present in animal tissues, such as hair, nails, horns^32^ or chitins are nitrogen-rich polysaccharides present in arthropods, and both are difficult to be degraded by microbes.^33^^,^^34^^,^^35^ Therefore, the different content and composition between animal- and plant-derived SOM could have different effects on soil carbon mineralization and sequestration.^25^^,^^36^

**Figure 1:**
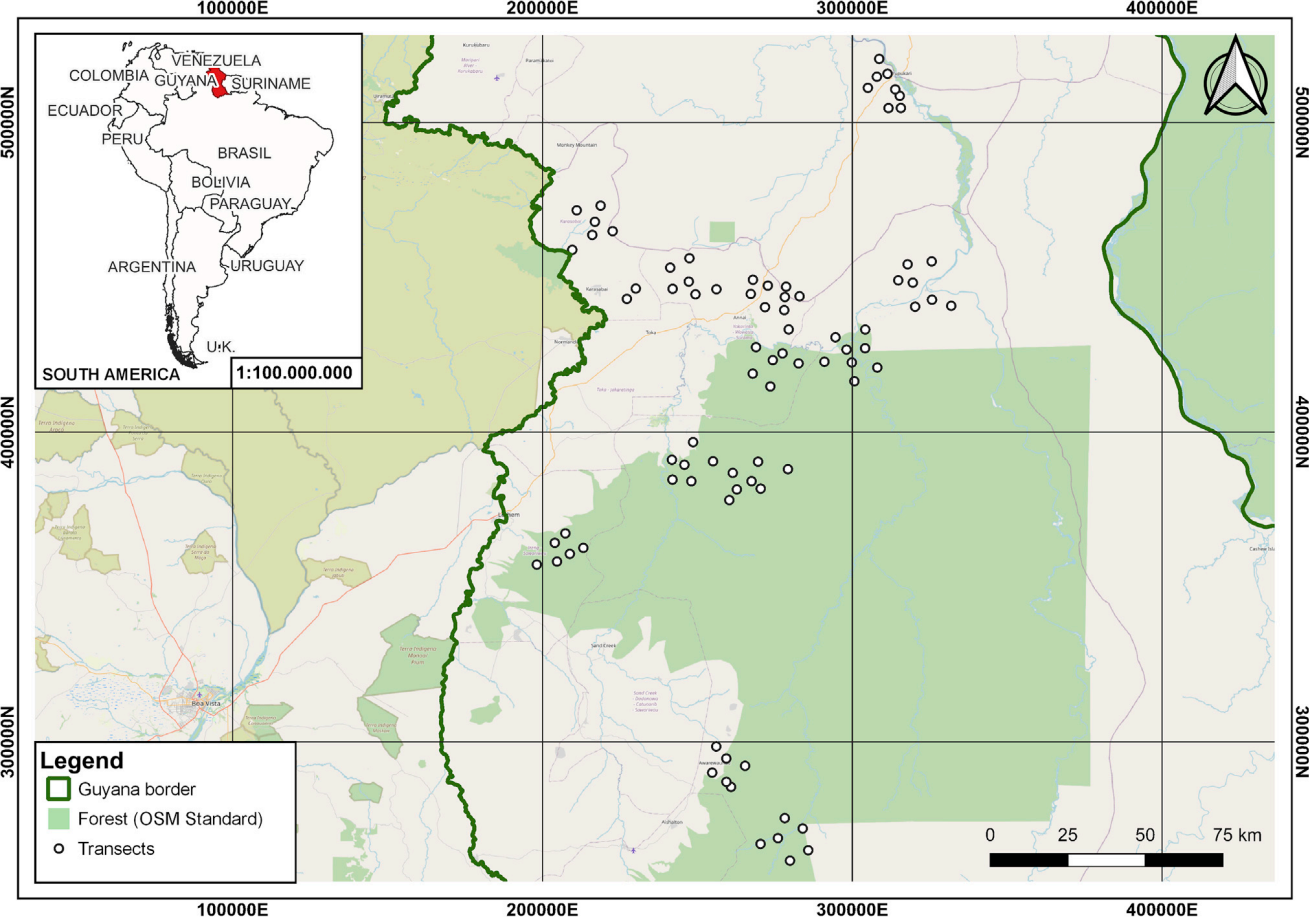
Study area Map of the study area in Guyana (WGS 84/UTM zone 21N), in northern South America (layer: http://www.diva-gis.org/Data, under Creative Commons Attribution 3.0 License), where the dots correspond to the transects surveyed (n = 83) in a forest-savannah system (layer: https://www.openstreetmap.org, ©OpenStreetMap, CC-BY-SA). This map was created with QGIS Desktop version 3.22.0 for Windows.^39^

Here, we investigate the effects of mammal and tree diversity on SOM composition at a large scale in the Amazon, an increasingly threatened megadiverse region that has historically acted as a carbon sink.^37^ Our study site encompasses 4.8 million hectares in the northern Amazon (Guyana). We analyzed the elemental and molecular composition of 401 soil samples, collected along 83 transects as a function of the species richness of 83 mammal and tree assemblages (Figure 1). We hypothesized that both SOM content and type accumulated would vary with mammal and tree richness. We also expected that mammal and tree community diversity would have differing effects on content and composition of SOM, because of differences between the inputs derived from plants versus animals. Clarifying the relationships between mammal diversity and the type of SOM accumulated in Amazonian soils will help us improve our understanding of the functioning of the carbon cycle and of the consequences of biodiversity loss for the capacity of soil carbon accumulation to mitigate climate change.^38^

## Results

We performed principal components analysis (PCA) by integrating the more common chemical elements (C, N, S, Fe, Al, Si) and infrared (IR) bands to assess main organic and inorganic groups in soils. This multivariate statistical analysis for quantitative data allows us to reduce the dimensionality of a large number of variables identifying the main soil organic and inorganic components and independently analyze covariation of SOM components in response to mammal and tree richness at community level while controlling for environmental heterogeneity among 83 transects.

### Soil composition

The elemental and molecular composition of the 401 soil samples was explained by three principal components, which accounted for 67.9% of total variance (Table S1). The first component was related to inorganic matter composition, whereas the second and third were related to organic matter composition, as follows: The first component (PC1 = 30.7%) showed large positive loadings for IR vibrations of secondary minerals (i.e., kaolinite and Fe-Al oxides). Large negative loadings in PC1 were found for quartz vibrations. Thus, this component describes a gradient of accumulation of clay minerals versus quartz content (Table S1, PC1). The second component (PC2 = 21.9%) is related to total SOM content. This component showed large positive loadings for carbon (C), nitrogen (N) and sulfur (S) concentrations, and positive loadings for aromatic and nitrogenated, carboxylated and aliphatic SOM, and total iron (Fe), aluminum (Al) and silicon (Si). Negative loadings were found for carbohydrates/silicates (Table S1, PC2). The third component (PC3 = 15.3%) essentially reflects differences in carbonyl and aliphatic SOM content versus N-, S-, Fe-rich SOM content. This component showed large positive loadings related to carboxylic/carboxylated and aliphatic compounds. Positive loadings were also found for aromatic compounds and carbohydrates. Negative loadings were obtained for total content of N, S, Fe, Al, and Si (Table S1, PC3).

### Mammal and tree richness effects on SOM composition

**Figure 2:**
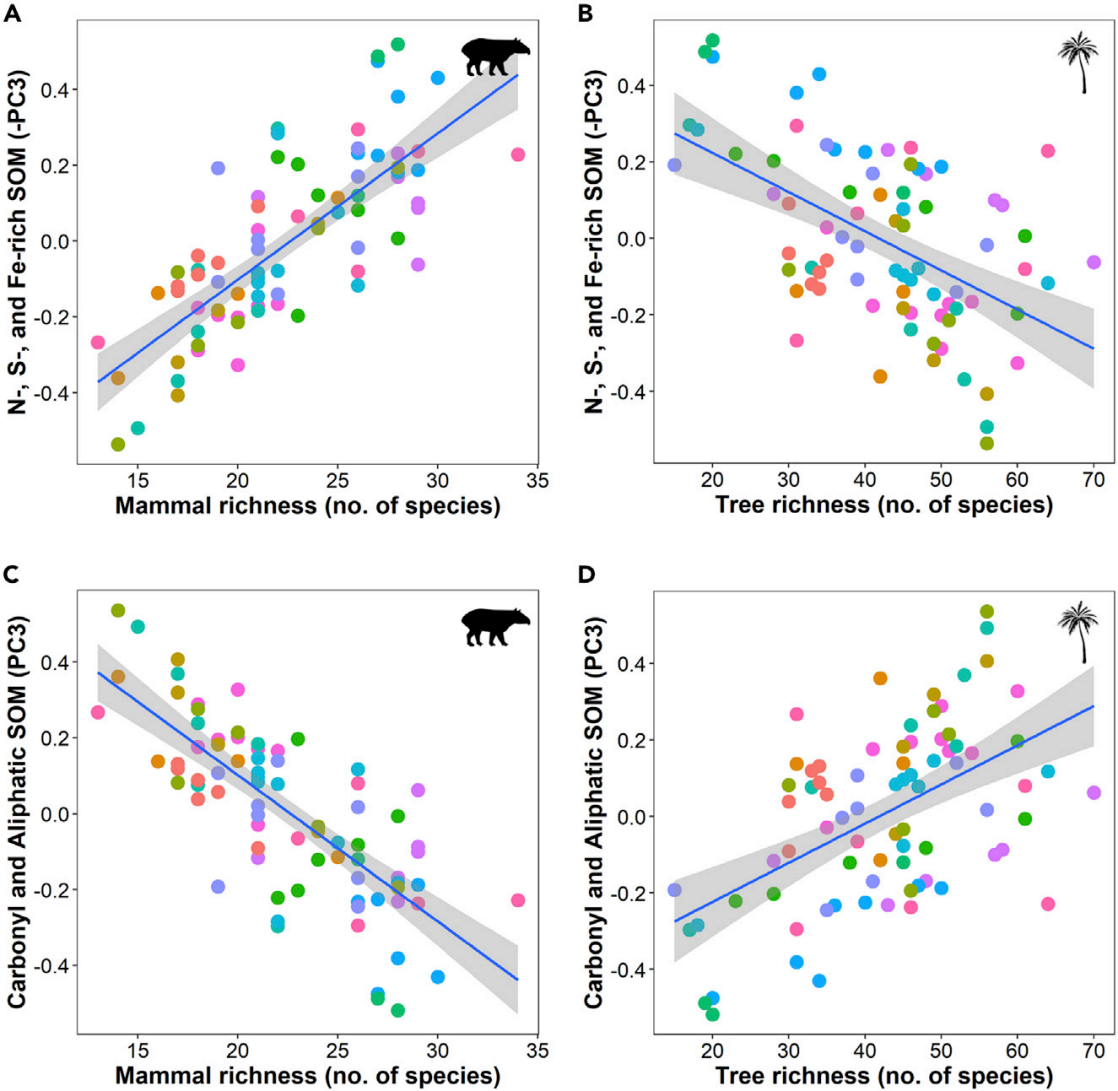
Mammal and tree richness effects on SOM composition LMM predicted values of N-, S-, Fe-rich SOM content (-PC3) and carbonyl and aliphatic SOM content (PC3) as a function of mammal richness (A and C) and tree richness (B and D) among 83 transects in the Amazon biome (transect arrays from each village are represented by different colored-dots).

We built Linear Mixed Models (LMM) for the three principal components which reflect inorganic component in PC1, total SOM content in PC2, different types of SOM composition in PC3 (Table 1). The LMM of the PC1 scores indicates that higher content of quartz is associated with areas with higher precipitation, whereas increased clay minerals content (i.e., kaolinite and iron-aluminum oxides) is associated with areas with higher tree richness (Table 1, PC1). The LMM of PC2 scores shows that total SOM content increased toward western areas and in more remote transects (distance to village and longitude effects, Table 1, PC2). The LMM of PC3 scores shows that mammal richness and tree richness have opposite effects on SOM composition. Carbonyl and aliphatic compounds covary negatively with N, S, Fe in these soils, and the effects of trees and mammals over this covariation are opposite to each other. The content of N-, S-, Fe-rich SOM increases with mammal diversity and decreases with tree diversity. The content of carbonyl and aliphatic SOM increases with tree diversity and decreases with mammal diversity (Table 1, PC3, Figure 2).

**Table 1:** Mammal and tree richness effects on soil composition

Response variable	Effects	Variance	SD	Estimate	s.e.	d.f.	Wald Chi-square	p-value
**Clay vs quartz content (PC1 =**30.71%**)**	**Precipitation**			−0.468	0.101	1	21.601	**<0.001**
n*=*83	**Tree richness**			0.170	0.076	1	4.999	**0.025**
*R*^*2*^_*m*_*=*0.313	Village (random)	0.057	0.240					
**Total SOM content (PC2 =**21.87%**)**	*Distance nearest village*			0.107	0.062	1	2.945	*0.086*
n*=*83	**Longitude**			0.386	0.150	1	6.669	**0.010**
*R*^*2*^_*m*_*=*0.220	Village (random)	0.270	0.519					
**Carbonyl and aliphatic vs N-, S-, Fe- rich SOM content (PC3 =15.32%)**	**Mammal richness**			−0.192	0.069	1	7.619	**0.006**
n*=*83	**Tree richness**			0.148	0.069	1	4.527	**0.033**
*R*^*2*^_*m*_*= 0.116*	Village (random)	5.198E-10	2.280E-05					

Results of Linear Mixed Models (LMMs) after reduction by minimum adequate model performed to analyze the effect of mammal and tree richness on the covariation of soil elemental and molecular composition among 83 transects from the Amazon biome (Guyana). In bold, all significant statistical effects (p < 0.05), and in italics, the marginal effects. Marginal coefficient of multiple determination (*R*^*2*^_*m*_) explain the amount of variation in each soil component (PC scores) explained by the fixed factors.

## Discussion

Mammal and plant diversity have been shown to influence key ecosystem functions, including carbon (C) storage.^6^^,^^40^^,^^41^ Our results are aligned with those findings and with recent work presenting evidence that mammals impact carbon stocks at landscape scale.^42^^,^^43^^,^^44^ However, although mammal and tree richness are positively linked to the soil carbon concentration in the northern Amazon,^8^ our analysis shows that they have opposite effects on soil organic matter (SOM) composition.

Previously, we had shown that the positive effect of both mammal and tree diversity on soil carbon concentration is mediated by the organic inputs generated through mammal feeding interactions with plants.^8^ We now find that mammal and tree diversity contribute to the accumulation of different types of SOM. The most remarkable finding is on the first hand, that the content of carbonyl (C=O bonds) and aliphatic (C-H bonds) compounds in SOM increases with the number of species in tree communities, and on the second hand, that nitrogen-, sulfur-, iron-rich SOM increases with mammal diversity. High mammal diversity also correlates with a higher concentration of iron (Fe) and aluminum (Al), which may be linked to a higher organo-mineral complexation in these soils.^45^ This result shows that tree and mammal diversity promote the accumulation of different components of SOM, thus their effects are not redundant, but rather they are complementary. In summary, more diverse mammal communities are contributing to increase N-, S-, Fe-rich SOM (SOM likely stabilized by organo-metal complexation), whereas species-rich tree communities are contributing to increase recalcitrant C-rich SOM (likely reducing SOM mineralization by microbes), both favoring soil organic carbon (SOC).

Mammal-mediated accumulation of N and S in soils is likely linked to animal inputs, e.g., carrion, hair, feces and urine.^10^^,^^29^^,^^32^^,^^46^ Besides, mammal diversity was related to higher contents of Fe and Al in soil, which may be because of organo-mineral associations that occur by co-precipitation of Al and Fe with organic compounds of dissolved SOM.^47^ A higher diversity of mammals might be contributing not only to the increase of the total N and S content in Amazonian soils, but also to a higher organo-metal complexation in soils with a higher total Fe and Al content,^45^^,^^48^ and thus, to the accumulation of more stable SOM, that means higher SOC retention. In contrast, tree richness is related to the accumulation of SOM rich in carbonyl groups and aliphatic compounds. It is known that plant diversity influences nutrient availability and soil fertility,^41^^,^^49^^,^^50^ depending on the different chemical composition of the inputs derived from plant species.^51^ SOC accumulation by plants is highly dependent on the accumulation of recalcitrant SOM which reduces carbon mineralization by microbes and enhances retention of atmospheric CO_2_ in soil.^25^

Organic remains are the main contributors to carbon concentration in soils at our study site, and they mainly accumulate through feeding interactions such as frugivory and carnivory.^8^ Large herbivores favor soil C by mediating N-stabilization of SOM^4^^,^^44^ and large frugivores increase soil ammonium and nitrates content.^28^ In contrast, large carnivores accelerate carrion consumption rates, favoring animal-derived SOM accumulation.^52^ Moreover, carnivore fecal matter shows a lower C:N ratio than that of frugivorous or herbivorous vertebrates with C:N ratios more similar to leaf litter.^9^ Thus, the type of SOM accumulated could also relate to the dominant trophic interactions in different communities^33^^,^^53^ affecting microbial metabolism and SOM decomposition,^9^ and thus, SOC accumulation.

Our results also indicate that tree diversity positively relates to soil inorganic composition, i.e., kaolinite and Fe-Al oxides content, contrary to the absent or negative correlations previously found in tropical systems.^54^^,^^55^^,^^56^ In tropical forests, certain trees and mycorrhizal fungi can release organic acid ligands and enzymes for nutrient uptake.^57^ If plants increase the content of free organic acids in SOM (i.e., carbonyl groups), they may contribute to soil acidification^58^ favoring a higher relative degree of mineral weathering, and thus, a higher formation of secondary minerals (i.e., kaolinite and Fe-Al oxides). Although soil inorganic composition was not the objective of this study, we accounted for the elements and dominant clay minerals in the inorganic fraction because they play a regulatory role in the accumulation and stabilization of SOM in tropical soils.^59^ In particular, OH groups of Fe-Al hydroxides contribute to retain organic molecules by forming metal-O-C bonds.^60^^,^^61^ Therefore, tree diversity seems to enhance secondary minerals accumulation that might favor stabilization of SOM, such as that accumulated by mammal diversity.

In summary, our study found that mammal and tree richness contribute to the accumulation of different types of SOM in the northern Amazon. We now know that the effect of mammals and trees on SOM is not redundant, but rather, it is complementary. Mammal diversity enhances the accumulation of SOM rich in N, S and Fe, and depleted in aliphatic and carbonyl compounds, which is the opposite effect that tree diversity has on SOM. Both, mammal and tree diversity seem to contribute in a complementary way to atmospheric carbon sequestration and storage in soils, potentially reducing CO_2_ in atmosphere.^62^ These results contribute to a still incomplete but rapidly evolving understanding of the carbon cycle in high-diversity tropical ecosystems and will help us better understand the role of organismal diversity in carbon cycle functioning, with implications for climate change mitigation.

### Limitations of the study

We found evidence of opposite effects of mammal and tree richness over soil organic matter (SOM) composition. However, the nature of our study is observational, and we do not have explicit tests for causality. Correlative studies are widely used to understand biodiversity effects on ecosystem functioning^63^^,^^64^^,^^65^ and often used to understand complex ecological processes that depend on multiple environmental factors at large spatial scales, such as carbon (C) and nitrogen (N) cycling.^8^^,^^66^ But long-term experimental enclosures could have improved the study,^4^^,^^28^^,^^44^ because they could have tested the causality of effects of mammal and tree contributions to SOM, and thus, to soil organic carbon (SOC) accumulation.

To estimate accumulation of carbon stocks it would have been necessary to measure depth and bulk density of soils as well as carbon content in different soil layers. Despite most current studies of biodiversity effects on soil C and N pools referring to soils up to 30 cm depth,^44^^,^^67^ carbon accumulation and persistence also vary with soil depth.^68^ Soil carbon content commonly decreases in deeper soils,^69^ but SOC stocks remain larger even up to 2 m depth.^70^ Therefore, the estimation of bulk density and carbon content at different soil depths would lead us to know the real impact of these mammal and tree communities on soil carbon storage.

Effects of diversity of mammal and tree communities on SOM composition may be related to the diversity of traits within different plant and animal communities.^14^^,^^71^^,^^72^ Indeed, trait diversity relates to ecosystem functioning even better than species richness.^73^^,^^74^ Future investigations should consider not only the number of species, but also their traits to better understand the role of mammal and tree communities in SOM composition and thus in SOC accumulation.

Last, given that microbial communities metabolize and decompose animal- and plant-derived organic remains accumulated in soil^10^^,^^75^ and that plant and animal species can impact on microbial activity and diversity differently,^11^^,^^76^ future work should consider microbial communities to look for a mechanistic relationship between mammals and trees and SOM composition. This is especially important because not all carbon molecules are degraded with the same efficiency by microbes and contribute to SOC accumulation.^22^^,^^77^

## STAR★Methods

### Key resources table

**Table:** 

REAGENT or RESOURCE	SOURCE	IDENTIFIER
**Software and algorithms**		

andurinha: Make Spectroscopic Data Processing Easier	Álvarez Fernández and Martínez Cortizas (2022)^78^	https://github.com/noemiallefs/andurinha
nlme: Linear and Nonlinear Mixed Effects Models	Pinheiro et al. (2002); Pinheiro and Bates (2000)^79^^,^^80^	https://CRAN.R-project.org/package=nlme
psych: Procedures for Psychological, Psychometric, and Personality Research	Revelle (2021)^81^	https://CRAN.R-project.org/package=psych
MASS: Support Functions and Datasets for Venables and Ripley’s MASS	Ripley and Venables (2002)^82^	https://www.stats.ox.ac.uk/pub/MASS4/
car: Companion to Applied Regression	Fox and Weisberg (2019)^83^	https://socialsciences.mcmaster.ca/jfox/Books/Companion/
R v4.2.1	http://www.r-project.org	RRID: SCR_001905
RStudio	http://www.rstudio.com/	RRID: SCR_000432
QGIS 3.22.0	http://www.qgis.org/	RRID: SCR_018507

**Other**		

XEPOS spectrometer	Ametek Corporation	https://www.ametek.com/
Mettler Toledo microbalance	Mettler Toledo	https://www.mt.com
Carlo Erba NA-1500 elemental analyzer	Thermo Scientific (Carlo Erba)	http://www.thermo.com
AGILENT CARY 630 FTIR spectrometer	Agilent Technologies Inc.	https://www.agilent.com/

### Resource availability

#### Lead contact

Further information and requests for any resources should be directed to and will be evaluated by the lead contact, José M. V. Fragoso (fragoso1@mac.com) to determine whether they can be made available on a case-by-case basis without identifiers.

#### Materials availability

This study did not generate new unique reagents or other materials.

### Experimental model and subject details

This work has not involved the use of human subjects or samples, nor has it use experimental models that require reporting of experimental model and subject details.

### Method details

#### Study area

This work was framed within a large-scale, participatory science project developed in the Rupununi region of southern Guyana (Figure 1).^8^^,^^84^ The study area includes 4,800,000 ha of tropical rainforest and rainforest-savannah transition across an environmental range of altitude (71–798 m.a.s.l.), longitude (198196.5E-331933.5E; WGS84/UTM21N), latitude (261634.5N–520568.0N; WGS84/UTM21N), temperature (23–27°C), annual precipitation (1,205–2,416 mm), and distance to the nearest village (1,040–12,593 m), road (592–51,375 m) and river (123–10,307 m). Like the rest of the Amazon, the area has high biological diversity, inlcuding 155 mammal species and 1,577 plant species,^85^ and is the home of Makushi, Wapishana and Wai-Wai indigenous people,^86^ who live at low-population levels (122–1,192 people per village)^84^ in remote areas (218–447 km distant from coastline), following traditional livelihood practices: hunting, fishing, and farming.^87^^,^^88^ Selective timber extraction occurs only for local use, and most habitats are well-preserved within the study area.^89^^,^^90^

#### Soil sampling

Sampling was performed along 83, 4 km-long straight-line transects spaced at least 3 km apart and centered around 13 villages, i.e., on average 6±1 transects per village, see Figure 1.^8^ A total of 401 soil samples (upper 10 cm after clearing surface litter) were collected in soil pits 20 cm in diameter and 10 cm deep excavated at different locations (3–8 pits per transect; mean ± SD: 6±2) along transects (Figure 1).

#### Mammal surveys

A total of 4,625 animal surveys were conducted by trained indigenous paratechnicians for 3 years (2007–2010) on all transects (56±17 surveys per transect); each transect was surveyed once a month for signs (tracks, feces, hair, carcasses, digging, burrows, eaten organic remains) in a 1-m-wide strip, and once a month for direct encounters,^91^^,^^92^ following standard distance sampling methods.^93^ A total 102,044 individuals of 48 mammal species (excluding volant mammals and small terrestrial mammals) recorded by direct visual encounters (19,347 individuals) and animal signs (82,697 individuals) were used for mammal species richness estimation.^8^

#### Tree surveys

Tree indentification included 24,552 individual trees with DBH >25 cm on 72 out of the 83 transects (July-December 2008). From a total of 163 taxa surveyed, 22% were identified only to the genus level, while the rest were identified to species level. All 83 transects were surveyed monthly for fruits and seeds on the ground (May 2007-June 2010). For 11 transects, tree richness was estimated using the relationship between fruit and tree richness on the rest of the transects (such as in Sobral et al.^8^).

#### Geochemical analyses (ED-XRF and CNH)

The quantitative elemental composition of 401 soil samples from 83 transects was analyzed by Energy Dispersive X-Ray Fluorescence (ED-XRF) spectrometry, using a XEPOS spectrometer (Ametek Corporation, Pennsylvania, USA). Spectrometer detects fluorescent X-rays emitted from a sample surface first excited by X-rays, differentiates the emissions with an analytical crystal, and determines the concentration of the main chemical elements (such as S, Fe, Al, Si considered for our work). This allows identification and quantification of the chemical composition of the soil samples.^94^ Each sample was first air-dried, sieved (<2 mm), ground finely (1.00–3.00 g) and compressed with a plunger (250–260 gm force) on a Teflon cup with a Prolene membrane at the bottom. The ED-XRF spectra obtained from 4 points from each cup were averaged and compared with the values obtained when measuring the certified standard reference material (NBS-1572; Gaithersburg MD). We considered the total content of Fe, Al, Si as elements which tend to accumulate under the intense weathering typical of tropical soils, both as resistant primary minerals (Si in quartz) and secondary phases (oxy-hydroxides of Fe and Al)^45^ but also N, S, as elements present in mineralized soil organic matter (SOM) of tropical soils and that can interfere with C accumulation during SOM formation.^95^ In addition, C and N concentrations (%) were determined as major elements of SOM on the same samples after they were oven-dried (110°C), sieved (<2 mm), weighed with a Mettler Toledo microbalance (+0.0005 mg) and analyzed by combustion using a Carlo Erba NA-1500 elemental analyzer.^8^ The total contents of C, N, S, Fe, Al, Si were Centered Log-Ratio (CLR) transformed to avoid the close data effect on compositional data prior to Principal Component Analysis (PCA).^96^

#### Infrared spectroscopy (FTIR-ATR)

Absorbance spectra of the 401 soil samples were obtained by Fourier Transform Infrared (FTIR) spectroscopy combined with Attenuated Total Reflectance (ATR), using an AGILENT CARY 630 FTIR spectrometer (Agilent Technologies Inc., California, USA). This technique allows us to identify the molecular composition of the soil from the characteristic molecular vibrations that are detected when a soil sample is subjected to mid-infrared radiation, and also to quantify the relative content of the main functional groups and organic compounds in SOM along with the mineral components of the soil inorganic fraction.^97^ We performed 100 scans per sample, between a wavelength range of 4000–400 cm^−1^ (mid-infrared region) and a spectral band resolution of ±4 cm^−1^. Sample spectra were processed and main peaks selected using the simple and fast routine provided by the {*andurinha*} R-package.^78^ This application provides standardized ("Z-score") spectra, the mean absorbance spectrum (the average of all absorbance spectra) and the standard deviation spectrum (to check for regions of the spectra with higher variability among samples). It also computes the second derivative spectra and determines the wavenumbers of the main absorbances and provides the standardized values for each band/peak and sample. For the characterization of the main inorganic soil components, we selected representative infrared (IR) bands of the main primary and secondary minerals: quartz (798 and 777 cm^−1^), kaolinite (3694, 3620, 911 cm^−1^) and iron-aluminum oxides/silicates (530 cm^−1^).^98^ For soil organic matter (SOM), total content is represented by total C, N and S (%); while the molecular composition of SOM is represented by functional IR groups: aromatic and nitrogenated SOM (1630 and 1550 cm^−1^), carboxylic/carboxylated SOM (1720, 1710 and 1700 cm^−1^) and aliphatic SOM (2920, 2850 cm^−1^).^99^^,^^100^^,^^101^ We therefore detect not only how much SOM is accumulated in these soils, but also whether the type of SOM accumulated is rich in C (aliphatic, carbonyl, and aromatic SOM) or in N (amides from proteins). Polysaccharides, a typical labile component of SOM, are not easy to identify in the spectra of mineral soils due to the C-O vibrations of the polysaccharides overlap with the Si-O vibrations of the silicate minerals.^102^ Additionally, tropical soils are usually depleted in plant-derived carbohydrates due to intense SOM transformation by microorganisms.^103^^,^^104^ To try to overcome these limitations, we also included vibrations at 1838 and 1823 cm^−1^, which may correspond both to mineral (i.e. silicates) vibrations and to the polysaccharides. The fractionation of their variance in the PCA enables us to discriminate between these two soil components, as well as the selected unrotated PCA solution.

### Quantification and statistical analysis

#### Soil composition

As indicated before, chemical elements and IR bands were selected to reflect the main chemical constituents of the studied soils, in particular regarding the total content (C, N, S) and composition of SOM (molecular organic compounds). Under the intense weathering conditions of the tropics, resistant minerals (i.e., quartz) and secondary minerals (i.e., clays) accumulate. Thus, we use total Fe, Al, and Si contents and IR band absorbances (i.e., kaolinite and Fe-Al oxides, and quartz) as proxies for resistant primary minerals and clay minerals. CLR transformed values of the 6 elements considered here (C, N, S, Fe, Al, Si) and the stardardized absorbances of 15 IR bands selected from FTIR-ATR analyses of the 401 soil samples were included in PCA,^105^ using the "principal" function of the {psych} R-package.^81^ In total, the first three principal components (PC) explained 68% of the total variance and the main signal of each PC was identified using the factor loadings (Table S1). Note that the PC scores obtained for the 401 soil samples were aggregated as the mean at the transect level (n = 83) to analyze the effects of mammal and tree richness on different types of SOM and inorganic composition obtained (Table S1).

#### Mammal and tree richness effects on SOM composition

We built three independent Linear Mixed Models (LMMs) using the "lme" function of the {nlme} R-package,^79^^,^^80^ where the response variable was the average scores of each of the three PCs aggregated at transect level (n = 83) with normal distribution and identity link function and the village closest to the transect as random factor. As fixed factors we included mammal richness per transect, tree richness per transect and the respective interaction. Additionally, to control the environmental heterogeneity among transects on soil composition variation, we included as fixed effects the distances to the nearest village, the nearest road and the nearest river (m), the longitude (X coordinate mean in m) and latitude (Y coordinate mean in m), the mean annual temperature (°C), and annual precipitation (mm), and the dominant lithology of each transect, defined by 8 classes: i) Continental sands and silts under thin Tertiary cover, ii) Gabbronorite sills and large dikes, iii) Granitoids including diorite, Makarapan riebeckite granite, pyroxene granite, iv) Granulites and charnockites, v) Greenstone belts mainly intermediate metavolcanics, vi) High grade gneisses, vii) Subvolcanic granites, viii) Acid or intermediate volcanics.^106^ All the continuous variables were standardized (mean = 0; SD = 1) to improve model convergence and for comparing the relative effects sizes of continuous predictors. For all analyses, the best minimum adequate model fitted by maximum likelihood (ML) was selected starting with a global model (containing all the cited covariables and the interaction term between mammal and tree richness) and then using the stepAIC() function of the {MASS} R-package^82^ for (both “backward” and “forward”) stepwise selection to remove the non-significant terms following the AICc criterium^107^ and final models were fitted by “REML” method. For all the models, we previously checked for multi-collinearity using the vif() function (VIF <10) of the {car} R-package.^83^ All statistical analyses were performed in RStudio environment^108^ for R version 4.2.1 for Windows.^109^

## Data Availability

• All data that can be publicly shared at this time are reported in this paper. Due to human rights concerns, and based on best practices for Free Prior Informed Consent (FPIC), the CARE Principles for Indigenous Data Governance, and respect for indigenous intellectual property rights, an agreement exists between the co-senior author and principal investigator of the project in the context of which biodiversity data and soil samples were collected (Project Fauna, José M. V. Fragoso (fragoso1@mac.com) and co-senior author, J.M.V.F.), and the project’s indigenous collaborators, that no raw or derived data can be publicly shared that could identify either communities or individuals; that raw and derived data use is restricted to publications and other uses that will not injure the territories, livelihoods, beliefs or intellectual property rights of the indigenous communities. • Original code generated in this paper is available from the José M. V. Fragoso (fragoso1@mac.com) upon request. • Any additional information required to reanalyze the data reported in this paper is available from the José M. V. Fragoso (fragoso1@mac.com) upon request.
