# Supplementary material for: Mammal and tree diversity accumulate different types of soil organic matter in the northern Amazon
Source: iScience. 2023 Jan 30;26(3):106088. doi: 10.1016/j.isci.2023.106088 (PMC10006633; doi:10.1016/j.isci.2023.106088)
Supplement: Document S1. Table S1 [file mmc1.pdf]

iScience, Volume *26*

Supplemental information

Mammal and tree diversity accumulate different types of soil organic matter in the northern Amazon

María Losada, Antonio M. Martínez Cortizas, Kirsten M. Silvius, Sara Varela, Ted K. Raab, Jose M. V. Fragoso, and Mar Sobral

SUPPLEMENTAL INFORMATION

Supplemental Tables

Table S1. Soil composition. Related to STAR Methods.
Factor loadings of the 3 first PCs extracted from PCA of soil elemental composition (total content of C, N, S, Fe, Al, Si with CLR transformation) and molecular composition (standardized mean absorbance of 15 IR bands selected as main inorganic and organic constituents, by their wavenumber - WN), and which explained a 67.91% of total variance (% explained by each PC in table) among 401 soil samples from 83 transects in Guyana.

**Soil composition**	**PC1[a] (30.71%)**	**PC2[b] (21.87%)**	**PC3[c] (15.32%)**
silicon (Si)	**-0.38**	**0.50**	**-0.36**
aluminum (Al)	**0.32**	**0.67**	**-0.38**
iron (Fe)	**0.69**	**0.37**	**-0.25**
iron-aluminum oxides/silicates (WN530)	**0.93**	0.11	-0.02
clay (kaolinite; WN911)	**0.89**	0.10	-0.04
clay (kaolinite; WN3620)	**0.89**	0.06	-0.12
clay (kaolinite; WN3694)	**0.91**	0.07	-0.06
quartz (WN777)	**-0.94**	-0.15	-0.07
quartz (WN798)	**-0.92**	-0.13	-0.03
carbon (C)	-0.07	**0.89**	-0.23
nitrogen (N)	0.07	**0.86**	**-0.29**
sulphur (S)	**-0.36**	**0.80**	**-0.31**
aromatic SOM (WN1550)	-0.13	**0.60**	**0.36**
aromatic-N SOM (WN1630)	-0.11	**0.63**	**0.29**
carbonyl SOM (carboxylates; WN1700)	-0.01	**0.36**	**0.71**
carbonyl SOM (organic acids; WN1710)	0.02	**0.26**	**0.69**
carbonyl SOM (carboxylic acids; WN1720)	0.14	0.17	**0.72**
aliphatic SOM (WN2850)	-0.17	**0.35**	**0.65**
aliphatic SOM (WN2920)	-0.1	**0.43**	**0.54**
carbohydrates/silicates (WN1823)	**0.52**	**-0.31**	**0.30**
carbohydrates/silicates (WN1838)	**0.49**	**-0.34**	**0.31**

Footnote1. Each PC represent different elemental-molecular soil composition, as follows:
[a] Clay *vs* quartz content (kaolinite + iron-aluminum oxides versus quartz)
[b] Total SOM content (C, N, S concentrations + aromatic-nitrogenated, carboxylated and aliphatic SOM)
[c] Carbonyl and aliphatic SOM *vs* N-, S-, Fe-rich SOM content (carbonyl groups + aliphaticity versus total N, S, Fe)
